# Influence of β_2_ adrenergic receptor genotype on risk of nocturnal ventilation in patients with Duchenne muscular dystrophy

**DOI:** 10.1186/s12931-019-1200-1

**Published:** 2019-10-16

**Authors:** Eli F. Kelley, Troy J. Cross, Eric M. Snyder, Craig M. McDonald, CINRG Investigators, Eric P. Hoffman, Luca Bello

**Affiliations:** 1 Department of Kinesiology, University of Minnesota, Minneapolis, MN USA; 2 Department of Cardiovascular Diseases, Mayo Clinic, RO_GE_MN_10, 1216 2nd Street SW, Rochester, MN 55902 USA; 3 University of California Davis Medical Center, Sacramento, CA USA; 4 Binghamton University – SUNY, Binghamton, NY USA; 5 Center for Genetic Medicine, Children’s Research Institute, Children’s National Health System, Washington, DC USA; 6 Department of Neurosciences, University of Padova, Padova, Italy

**Keywords:** Duchenne muscular dystrophy, beta2-adrenergic receptor, Genotype, Respiratory, Nocturnal ventilation, Risk

## Abstract

Duchenne muscular dystrophy (DMD) is a progressive neuromuscular disease resulting in severe respiratory derangements. As such, DMD patients are at a high risk of nocturnal hypoventilation, thereby requiring nocturnal ventilation (NV). To this end, NV is an important clinical milestone in the management of DMD. Emerging evidence suggests that ß_2_ adrenergic receptors (ADRB2) may play a role in determining respiratory function, whereby more functional ADRB2 genotype variants (e.g., Gly16) are associated with improved pulmonary function and respiratory muscle strength. These findings suggest that the more functional ADRB2 genotype may help to preserve respiratory function in patients with DMD. The purpose of this study was to identify the influence of ADRB2 genotype on the risk of NV use in DMD. Data from the CINRG Duchenne Natural History Study including 175 DMD patients (3–25 yrs) were analyzed focusing on ADRB2 genotype variants. Time-to-event analyses were used to examine differences in the age at prescription of full-time NV use between genotypes. There were no differences between genotype groups in age, height, weight, corticosteroid use, proportion of ambulatory patients, or age at loss of ambulation. DMD patients expressing the Gly16 polymorphism had a significantly (*P* < 0.05) lower mean age at NV prescription compared with those patients expressing the Arg16 polymorphism (21.80 ± 0.59 yrs. vs 25.91 ± 1.31 yrs., respectively). In addition, a covariate-adjusted Cox model revealed that the Gly16 variant group possessed a 6.52-fold higher risk of full-time NV use at any given age compared with the Arg16 polymorphism group. These data suggest that genetic variations in the ADRB2 gene may influence the age at which DMD patients are first prescribed NV, whereby patients with the Gly16 polymorphism are more likely to require NV assistance at an earlier age than their Arg16 counterparts.

## Background

It is estimated that 32% of Duchenne muscular dystrophy (DMD) patients suffer from nighttime alveolar hypoventilation as a result of sleep-disordered breathing [1]. Sleep-disordered breathing in this population is a result of respiratory weakness, and is associated with cardiac morbidity, neurocognitive deficits, and impaired lung function [1, 2]. As respiratory muscle weakness progresses, it results in the inability to generate a sufficient respiratory pressure to maintain adequate alveolar ventilation, leading to constant alveolar hypoventilation [3]. In patients with DMD, sleep-disordered breathing tends to precede daytime hypoventilation, which is a significant contributor to hospitalization and mortality rates [4]. The age at first use of nocturnal ventilation (NV) is therefore considered an important clinical milestone in these patients, and indicates significant respiratory derangement [1, 5]. It is therefore important to understand the factors which affect the risk of nocturnal hypoventilation, such that we may identify novel therapeutic interventions to delay dependence on NV, and offset the progression to day-time ventilator assistance, ultimately decreasing hospitalization and mortality rates, and improving quality-of-life.

Respiratory muscle weakness in DMD is characterized by a progressive loss in the ability to generate respiratory pressures, resulting in severe ventilatory derangements [6, 7]. Decreases in respiratory pressure and airflow generation can be attributed to a primary weakness of the diaphragm secondary to intramuscular remodeling in DMD [6]. This remodeling is characterized by an increased resting diaphragm thickness due to an infiltration and deposition of non-contractile elements (pseudo-hypertrophy), concomitant with the loss of sarcomeres in series [8, 9]. These dystrophic alterations together adversely affect the length-tension relationship and contractility of the diaphragm [8]. Due to the progressive nature of respiratory weakness in DMD, it is imperative that therapeutic targets are identified to slow respiratory muscle degradation, and thus decrease the risk of NV prescription.

One novel pathway that has been identified as capable of attenuating skeletal muscle degradation in DMD is the β_2_-adrenergic receptor (ADRB2) coupled pathway [10–12]. β_2_-adrenergic receptors influence bulk muscle size, strength and regeneration [13, 14]. ADRB2 stimulation has also been shown to: (i) increase diaphragmatic cross-sectional area, strength, and contractility [11, 12, 15]; (ii) improve mucocilliary clearance [16–18]; (iii) inhibit inflammatory pathways [19–23]; and to (iv) inhibit calpain activity and decrease its concentration [24–28]. In light of the above, the functionality of ADRB2 may play a significant role in the protection of respiratory muscles from dystrophic pathways, and provide a novel therapeutic target in DMD to delay the dependence on NV.

The single nucleotide polymorphism (SNP) rs1042713, situated within the coding sequence of *ADRB2*, causes the substitution of an Arginine (Arg) with a glycine (Gly) at amino acid position 16 of the receptor, improving its functionality. In fact, the Gly16 polymorphism is globally expressed, has a higher receptor density, and is more resistant to receptor down-regulation than its Arg16 counterpart [29, 30]. The Gly16 polymorphism is associated with sustained bronchodilation at rest and following intense exercise, and improved lung function in healthy pediatric patients and patients with heart failure [31–33]. The association between the Gly16 polymorphism and improved lung function in healthy and diseased populations suggests a possible therapeutic target for preserving respiratory function and delaying NV dependence in DMD patients. However, to date, no studies have investigated the influence of *ADRB2* genotype on respiratory insufficiency in this clinical population.

The aim of this study was to examine the influence of *ADRB2* genotype on the risk of full-time NV prescription in patients with DMD. Given that ADRB2 stimulation may provide several benefits to respiratory function (e.g., increased diaphragmatic contractility, improved mucocilliary clearance, inhibited inflammatory pathways and calpain activity), we hypothesized that DMD patients with the more “functional” ADRB2 polymorphism (i.e., Gly16) would demonstrate a lower risk of NV use compared with DMD patients expressing the less functional polymorphism (i.e., Arg16).

## Methods

### Participants

Data analyzed for this study were a part of a larger dataset from the Cooperative International Neuromuscular Research Group Duchenne Natural History Study (CINRG-DNHS). All participants included in this study and/or their legal guardians consented specifically to genotyping of genetic variants for research purposes, and the study was approved by local institutional or ethics review boards at each participating institution. One-hundred seventy-five patients with a clinical diagnosis of DMD (ages 3–25 years at entry into the study) were identified and included in the dataset used in this study. Patients were followed for a maximum of 9.7 years. This study focused on a functional *ADRB2* protein-altering variant at codon 16 employing an Exome Chip (a genotyping chip focused on variants situated within gene-coding portions of the genome). Exome Chip genotyping and data cleaning methods in the CINRG-DNHS cohorts have been previously described [34].

### Data analyses

Patient age, height, weight, corticosteroid use, ambulatory status, and forced vital capacity (FVC) at entry into the study were obtained from the CINRG-DNHS database. All reported patient heights were calculated from ulnar length, as described by others [35]. Corticosteroid use was derived from clinically reported start and stop dates. Nocturnal ventilation status was determined by the clinic visit at which full-time NV (e.g., Bi-PAP mask, *n* = 40; Bi-PAP nasal pillows, *n* = 9; C-PAP, *n* = 3; and mouthpiece, *n* = 3) was first prescribed to the patient.

### Statistical analyses

Group demographics were compared using a one-way analysis of variance (ANOVA). To investigate differences among the specific genotype groups, a Tukey honest significant difference (HSD) post-hoc comparison was used. Genotype differences in the risk of NV use at any given age were estimated by a Kaplan-Meier analysis and the log-rank test. A Cox proportional hazard (PH) model was used to examine the effects of genotype variant on the risk of NV in DMD patients, after adjusting for patient age, weight, ambulatory status, corticosteroid use, and FVC. Time dependence of the covariates was ruled out after inspection of the Schoenfeld residuals scores [36]. We chose to group *ADRB2* genotypes according to a dominant model for the Gly allele, i.e. homozygous for Arg at amino acid spot 16 (henceforth named “Arg16 group”, *n* = 26) vs. homozygous or heterozygous for Gly at amino acid 16 (“Gly16 group”, *n* = 149) because preliminary analyses of the data demonstrated phenotypic similarity of heterozygotes with Gly homozygotes. Furthermore, this dominant model is consistent with previous literature [32, 33, 36]. For all covariates entered into the Kaplan-Meier and Cox PH analyses, outliers were identified as observations above or below the 95th percentile for residual deviance score. Additional observations were removed from the analysis if they did not have an FVC within 6 months of study entry or were already on NV at study entry (i.e. left-censored). All statistical comparisons were made using a statistical software packages (RStudio; RStudio Inc., Boston, MA, USA, version 1.1.456). Statistical analyses were considered significant if *P* < 0.05.

## Results

### Subject characteristics

Genotypes at rs1042713 were as follows: AA (Arg/Arg) *n* = 26, AG (Arg/Gly) *n* = 90, and GG (Gly/Gly) *n* = 59. This genotype distribution does not violate Hardy-Weinberg equilibrium, and the observed minor allele frequency of 0.41 is close to that reported in global populations (0.42 reported in ExAC). There were no differences in age, height, weight, corticosteroid use, number of ambulatory patients, or age of loss of ambulation between genotype groups (Table 1). Furthermore, there was no difference in age at loss of ambulation between Gly16 and Arg16 genotype groups (10.96 ± 0.23 yrs. vs 11.93 ± 0.75 yrs., respectively). A total of 78 patients were non-ambulatory at entry into the study, consisting of 65 patients in the Gly16 group, and 13 patients in the Arg16 group (72 and 50% of the genotype sample populations, respectively, χ^2^ *p* = 0.058).

**Table 1:** Subject characteristics

	Mean	SE	*p-value*
Age (yrs)			
*Arg16*	12.97	1.23	0.61
*Gly16*	12.34	0.47	
*Total*	12.43	0.44	
Height (cm)			
*Arg16*	138.85	4.27	0.62
*Gly16*	141.22	1.82	
*Total*	140.87	1.67	
Weight (kg)			
*Arg16*	42.91	4.12	0.98
*Gly16*	43.01	1.89	
*Total*	42.99	1.71	
Corticosteroid Use (yrs)			
*Arg16*	3.47	0.72	0.93
*Gly16*	3.39	0.34	
*Total*	3.40	0.31	

*SE* standard error; Arg16: patients who were homozygous or heterozygous for the β_2_-adrenergic receptor (ADRB2) resulting in at least one arginine substitution at amino acid 16 (*n* = 26); Gly16: patients who were homozygous for ADRB2 resulting in a glycine substitution at amino acid 16 (*n* = 149)

### Kaplan-Meier analysis

**Fig. 1:**
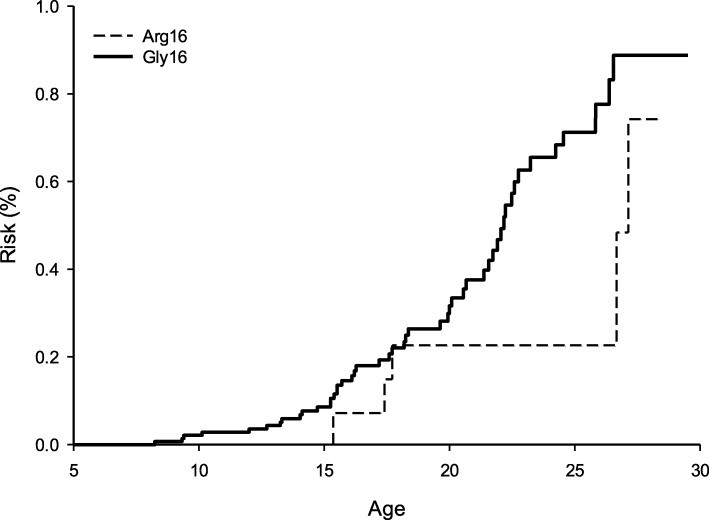
The cumulative risk of NV in DMD patients stratified by ADRB2 genotype. The cumulative risk of nocturnal ventilation in DMD patients was calculated from the survival curve produced by the Kaplan-Meier analysis

Two observations were identified as outliers in a preliminary time-to-event analysis of age at NV dependence, and were omitted from further analyses (173 observations remaining). The results of the Kaplan-Meier analysis are illustrated in Fig. 1. The mean and median age at first use of NV are presented in Table 2. DMD participants with the Gly16 polymorphism demonstrated a higher risk (*P* < 0.05) of use of NV at any given age compared with those expressing the Arg16 polymorphism (21.80 ± 0.59 yrs. vs 25.91 ± 1.31 yrs., respectively).

**Table 2:** Kaplan-Meier mean and median ages at first use of NV in patients with DMD

	Mean Age			Median Age		
	Estimate (yrs)	SE	95% CI	Estimate (yrs)	SE	95% CI
Arg16	25.91*	1.31	23.35–28.49	28.30*	4.14	20.19–36.41
Gly16	21.80	0.59	20.86–23.42	22.17	0.40	21.38–22.96
Overall	22.71	0.62	21.49–23.93	22.48	0.39	21.72–23.24

*SE* standard error, *CI* confidence interval, Arg16: patients who were homozygous or heterozygous for the β_2_-adrenergic receptor (ADRB2) resulting in at least one arginine substitution at amino acid 16 (*n* = 26); Gly16: patients who were homozygous for ADRB2 resulting in a glycine substitution at amino acid 16 (*n* = 147). *Significant difference in mean or median age at first use of NV between genotype groups, *P* < 0.05

### Cox proportional Hazard

**Fig. 2:**
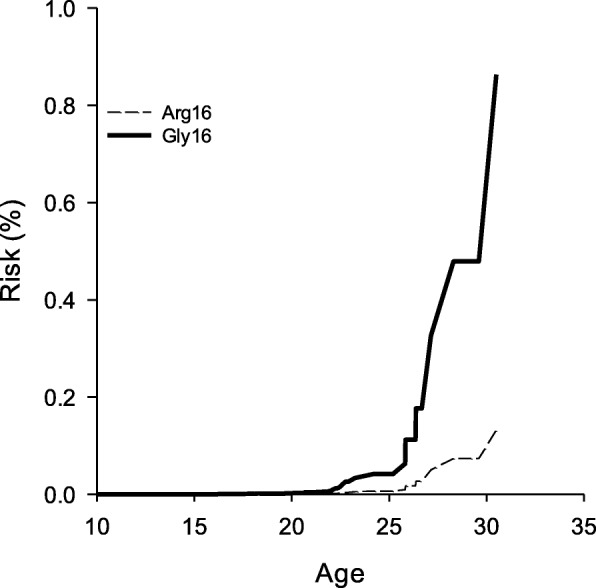
The cumulative risk of NV in DMD patients stratified by ADRB2 genotype. The cumulative risk function was obtained from Cox regression modeling of the risk of nocturnal ventilation where genotype group, patient age at entry into the study, ambulatory status, corticosteroid-use, weight, and FVC were entered into the model as covariates. The risk curves was produced for each genotype variant by holding all other covariates in the Cox model constant at their respective means (Age = 12.2 yrs.; Mass = 43.1 kg; Corticosteroid-use = 3.4 yrs.; Ambulatory status = 0.60; FVC = 1.71)

Thirty-one observations were omitted from further analyses (144 observations remaining). These observations were identified as outliers (*n* = 9), as not having an FVC measure (*n* = 7), or being on full-time NV use at entry into the study (i.e. left-censored, *n* = 15) in a preliminary analysis of the Cox PH model. The results of the Cox PH analysis are presented in Table 3. Patient age at study entry, ambulatory status, and FVC were strong, negative predictors of the risk of NV assistance in patients with DMD at any given age (*P* < 0.05). In contrast, *ADRB2* genotype was identified as a strong, *positive* predictor of NV use in DMD patients (*P* < 0.05). Specifically, those DMD participants with the Gly16 genotype variant were approximately 6.52 times more likely to be given NV assistance at any given age than those with the Arg16 polymorphism. Patient weight was also identified as a positive predictor of full-time NV use (*P* < 0.05). The cumulative risk of NV use in DMD patients stratified by ADRB2 genotype are presented in Fig. 2.

**Table 3:** Cox regression analysis of the NV risk at any given age in patients with DMD

	β	SE	Wald	*p-value*	HR	95% CI
Genotype	1.87	0.71	2.66	< 0.05	6.52	1.64–25.99
Age (yrs)	−0.57	0.10	−5.55	< 0.05	0.57	0.46–0.69
Ambulatory Status	**−**3.01	0.79	−3.84	< 0.05	0.05	0.01–0.23
Corticosteroid-use (yrs)	**−**0.07	0.05	−1.44	0.15	0.93	0.84–1.03
Weight (cm)	0.02	0.01	2.29	< 0.05	1.02	1.00–1.04
FVC (L)	−1.80	0.45	−4.02	< 0.05	0.17	0.07–0.39

*SE* standard error, *HR* hazard ratio, *CI* confidence interval, *FVC* forced vital capacity, Age: patient age at entry into the study; Arg16: patients who were homozygous or heterozygous for the β_2_-adrenergic receptor (ADRB2) resulting in at least one arginine substitution at amino acid 16 (*n* = 26); Gly16: patients who were homozygous for ADRB2 resulting in a glycine substitution at amino acid 16 (*n* = 147); genotype was coded as: 0 = Arg16, 1 = Gly16; ambulatory status was coded as: 0 = non-ambulatory, 1 = ambulatory; there was a significant influence of genotype, ambulatory status, weight, and FVC on risk of NV use

## Discussion

The original hypothesis of this study was that DMD patients with the Gly16 polymorphism would have a reduced risk of using NV at any given age compared with those patients expressing the Arg16 polymorphism. The rationale for this hypothesis lay on the basis that ADBR2 stimulation may: increase diaphragmatic cross-sectional area, strength, and contractility [11, 12, 15]; improve mucocilliary clearance [16–18]; inhibit inflammatory pathways [19–23]; and inhibit calpain activity and decrease concentration [24–28]. As such, it was expected that possessing the “functional” ADBR2 genotype (i.e., Gly16) would confer a positive effect on retarding the progressive loss of respiratory function and, by extension, delaying the age at dependence on NV in DMD patients. Our results did not support this hypothesis. Instead, the present study demonstrated that DMD patients with the Gly16 polymorphism display an approximate 6.5-fold per-year *increase* in the risk of becoming dependent on NV than those with the Arg16 variant. We propose two potential explanations for these unexpected findings: that the Gly16 polymorphism may (i) increase the rate of contraction-induced injuries and (ii) may increase intracellular [Ca^2+^].

ADRB2 stimulation increases myofibril expression of type IIa myosin heavy chain isoform in animal models, promoting a fiber-type shift from slow-oxidative to more fast-glycolytic muscle fibers [37, 38]. Type II fibers have an increased peak contractile strength compared with type I fibers [39]. Indeed, healthy adults expressing the Gly16 polymorphism often display increased muscular strength and power relative to their Arg16 counterparts [36]. While it may seem at first that increased strength is beneficial for respiratory muscle fatigue and weakness, the augmentation of contractile force likely increases the number, and rate of contraction-induced injury in DMD patients. Dystrophin-deficient muscle fibers lack adequate membrane stability to efficiently distribute forces associated with myofibril contraction across the sarcolemma [40]. As a result, dystrophin-deficient muscle fibers are highly susceptible to contraction-induced injuries — a significant component of muscle fiber degradation in DMD [41]. Further, the type I to type II fiber type shift renders the muscle fiber more susceptible to eccentric contraction-induced damage, and increased fatigability [42, 43]. In light of the above, it is at least conceivable that the Gly16 polymorphism, by increasing the rate of contraction-induced injuries, hastens the progression of respiratory muscle weakness in DMD patients, leading to a higher risk of NV use.

A secondary explanation for our current findings is that DMD patients with the Gly16 polymorphism, by virtue of increased ADRB2 activity [29, 30], may have incurred additional skeletal muscle damage due to relatively higher intracellular concentrations of Ca^2+^ ([Ca^2+^]). In murine models, ADRB2 stimulation enhances Ca^2+^ efflux from the sarcoplasmic reticulum via a G-protein linked pathway [44], promoting a rise in intracellular [Ca^2+^]. This facilitatory effect of ADRB2 stimulation on rising intracellular [Ca^2+^] is amplified by the abnormally low gene expression of regucalcin in DMD patients, i.e., the principal Ca^2+^-binding protein in the diaphragm [45]. Further to the above, the potentially higher rate of contraction-induced injuries in DMD patients with the Gly16 polymorphism (see above) may promote further Ca^2+^ influx into the cytosol from the extracellular space [46]. It is emphasized that increased intracellular [Ca^2+^] promotes calpain activity, upregulating proteolytic cellular damage and myofibril degradation [44, 45]. As such, we speculate that the Gly16 polymorphism is associated with poorer intracellular [Ca^2+^] homeostasis and greater calpain activity, the result of which may hasten the onset of respiratory muscle weakness, leading to a higher risk of NV use than those patients expressing the Arg16 polymorphism.

### Implications of our findings

**Fig. 3:**
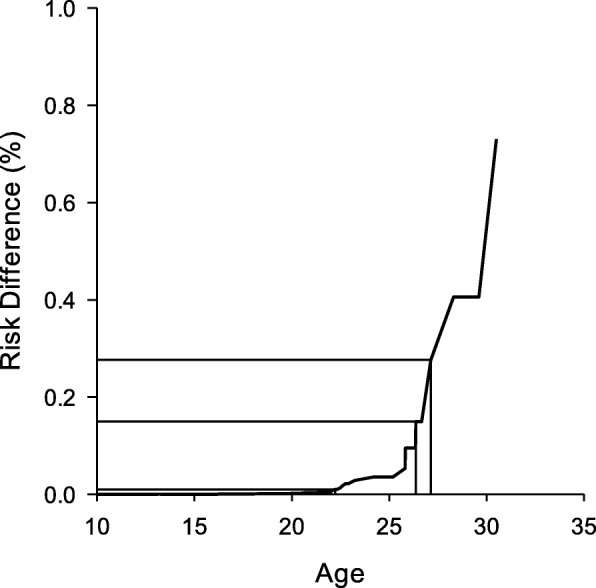
The absolute difference in risk of NV in DMD patients stratified by ADRB2 genotype. The risk curve is representative of the absolute difference in the cumulative risk for each genotype. The cumulative risks for each genotype was obtained from the Cox regression modeling of the risk of nocturnal ventilation where genotype group, patient age at entry into the study, ambulatory status, corticosteroid-use, weight, and FVC were entered into the model as covariates. The reference lines denote the absolute difference in risk between genotype variants at the ages of 22 and 27 years of age. Note that the difference in risk of NV between Arg16 and Gly16 genotype groups is marginal (< 1%) at ages below 22, yet widens greatly to over 25% by the age of 27 years

Pilot studies have shown an improvement in isometric knee-extensor strength and manual muscle test scores in DMD patients following 12 weeks of ADRB2 agonist treatment [47]. A larger follow-up study also demonstrated that ADRB2 agonist treatment improves lean body mass and time to walk/run 30 ft. in patients with DMD [48]. Importantly, however, both study populations consisted of young DMD patients whose ages ranged between 5 to 11 years. We emphasize here that although our findings demonstrate that the Gly16 polymorphism confers a higher risk of NV use at any given age, the absolute difference in absolute risk between Gly16 and Arg16 groups was marginal for preadolescent and adolescent patients (Fig. 3). Indeed, the absolute risk difference between genotype groups was less than 1% up to 22 years of age in our cohort of DMD patients. The difference in risk of NV between groups widened thereafter to reach > 25% at patient ages of 27 years and older. One may infer from these observations that the increase in risk of NV use in the Gly16 compared with the Arg16 polymorphism is relatively trivial below the adolescent years, and becomes more important when DMD patients enter the second decade of life. Further studies are needed to examine whether the effects of the Gly16 polymorphism on respiratory outcomes in DMD are indeed dependent on patient age.

### Other factors influencing risk of nocturnal ventilation in DMD

Our Cox regression analysis demonstrated that patient age at entry into the study, ambulatory status, weight, and FVC were also significant predictors of the risk of NV in DMD patients. Firstly, it is expected that FVC should confer a protective (i.e., negative) effect on the risk of NV use in DMD as FVC decline is strongly correlated with the onset of nocturnal hypoventilation and the need for NV assistance in patients with DMD [49]. In fact, FVC is the primary clinical measure of respiratory function in this population and is often used as a metric for the need for NV prescription [49]. Secondly, it is not surprising that ambulatory status decreased the risk of NV in our cohort, seeing that DMD patients who are ambulatory typically display a better clinical prognosis [50]. Thirdly, patient weight was also identified as a significant, negative risk factor of NV – an expected finding given that weight is negatively associated with respiratory function and ambulatory status in this population [49]. And finally, patient age at entry into the study was also a negative predicator of the risk of NV use. This may be due to a number of patients who were not prescribed NV during the study follow-up period were also older at entry into the study. One explanation may be these patients having entered into the study at a later age as a result of being healthier and living longer without severe respiratory weakness or derangement.

### Methodological considerations

It is emphasized that data for this study was collected prior to the publication of the 2018 DMD Care Considerations and the DNHS protocol did not specify when clinicians had to implement NV (e.g. at a certain FVC, pCO2, or nocturnal desaturation event threshold). Therefore, the clinically adopted thresholds may vary between clinics [49]. However, recommendations for the monitoring of sleep quality, symptoms of sleep-disordered breathing, and annual polysomnography with continuous CO_2_ to detect nighttime hypoventilation and the need for NV precede the current DMD Care Considerations [51]. As such, it may be assumed patients were properly monitored and that clinical standards of care were similar between populations.

## Conclusions

The findings of the present study indicate that DMD patients with the Gly16 ADRB2 polymorphism are approximately 6.5 times more likely to require full-time NV assistance at a given age compared with those patients expressing the Arg16 polymorphism. Despite prior speculation that ADRB2 stimulation may be a useful therapeutic approach, our data suggest caution in using ADRB2 agonist treatment for post-adolescent DMD patients at risk for nocturnal hypoventilation.

## Data Availability

The data that support the findings of this study are available from CINRG but restrictions apply to the availability of these data, which were used under license for the current study, and so are not publicly available. Data are however available from the authors upon reasonable request and with permission of CINRG.

## Abbreviations

ADRB2: Beta-2 adrenergic receptor
ANOVA: Analysis of variance
Arg: Arginine
Ca^2+^: Calcium
CINRG-DNHS: Cooperative International Neuromuscular Research Group Duchenne Natural History Study
DMD: Duchenne muscular dystrophy
FVC: Forced vital capacity
Gly: Glycine
HSD: Honest significant difference
NV: Nocturnal ventilation
PH: Proportional hazard
